# Estimated Number of COVID-19 Infections, Hospitalizations, and Deaths Prevented Among Vaccinated Persons in the US, December 2020 to September 2021

**DOI:** 10.1001/jamanetworkopen.2022.20385

**Published:** 2022-07-06

**Authors:** Molly K. Steele, Alexia Couture, Carrie Reed, Danielle Iuliano, Michael Whitaker, Hannah Fast, Aron J. Hall, Adam MacNeil, Betsy Cadwell, Kristin J. Marks, Benjamin J. Silk

**Affiliations:** 1 COVID-19 Emergency Response, Centers for Disease Control and Prevention, Atlanta, Georgia; 2 Division of Viral Diseases, National Center for Immunization and Respiratory Diseases, Centers for Disease Control and Prevention, Atlanta, Georgia; 3 Influenza Division, National Center for Immunization and Respiratory Diseases, Centers for Disease Control and Prevention, Atlanta, Georgia; 4 US Public Health Service, Rockville, Maryland; 5 Immunization Services Division, National Center for Immunization and Respiratory Diseases, Centers for Disease Control and Prevention, Atlanta, Georgia; 6 Epidemic Intelligence Service, Centers for Disease Control and Prevention, Atlanta, Georgia; 7 Division of Nutrition, Physical Activity, and Obesity, National Center for Chronic Disease Prevention and Health Promotion, Atlanta, Georgia

## Abstract

**Importance:**

The number of SARS-CoV-2 infections and COVID-19–associated hospitalizations and deaths prevented among vaccinated persons, independent of the effect of reduced transmission, is a key measure of vaccine impact.

**Objective:**

To estimate the number of SARS-CoV-2 infections and COVID-19–associated hospitalizations and deaths prevented among vaccinated adults in the US.

**Design, Setting, and Participants:**

In this modeling study, a multiplier model was used to extrapolate the number of SARS-CoV-2 infections and COVID-19–associated deaths from data on the number of COVID-19–associated hospitalizations stratified by state, month, and age group (18-49, 50-64, and ≥65 years) in the US from December 1, 2020, to September 30, 2021. These estimates were combined with data on vaccine coverage and effectiveness to estimate the risks of infections, hospitalizations, and deaths. Risks were applied to the US population 18 years or older to estimate the expected burden in that population without vaccination. The estimated burden in the US population 18 years or older given observed levels of vaccination was subtracted from the expected burden in the US population 18 years or older without vaccination (ie, counterfactual) to estimate the impact of vaccination among vaccinated persons.

**Exposures:**

Completion of the COVID-19 vaccination course, defined as 2 doses of messenger RNA (BNT162b2 or mRNA-1273) vaccines or 1 dose of JNJ-78436735 vaccine.

**Main Outcomes and Measures:**

Monthly numbers and percentages of SARS-CoV-2 infections and COVID-19–associated hospitalizations and deaths prevented were estimated among those who have been vaccinated in the US.

**Results:**

COVID-19 vaccination was estimated to prevent approximately 27 million (95% uncertainty interval [UI], 22 million to 34 million) infections, 1.6 million (95% UI, 1.4 million to 1.8 million) hospitalizations, and 235 000 (95% UI, 175 000–305 000) deaths in the US from December 1, 2020, to September 30, 2021, among vaccinated adults 18 years or older. From September 1 to September 30, 2021, vaccination was estimated to prevent 52% (95% UI, 45%–62%) of expected infections, 56% (95% UI, 52%-62%) of expected hospitalizations, and 58% (95% UI, 53%-63%) of expected deaths in adults 18 years or older.

**Conclusions and Relevance:**

These findings indicate that the US COVID-19 vaccination program prevented a substantial burden of morbidity and mortality through direct protection of vaccinated individuals.

## Key Points

**Question:**

How many SARS-CoV-2 infections and COVID-19–associated hospitalizations and deaths have been prevented among vaccinated persons by the US COVID-19 vaccination program?

**Findings:**

In this modeling study, COVID-19 vaccination was estimated to prevent 27 million SARS-CoV-2 infections, 1.6 million COVID-19–associated hospitalizations, and 235 000 COVID-19–associated deaths among vaccinated persons 18 years or older from December 1, 2020, to September 30, 2021. By September 30, 2021, vaccination prevented an estimated 52% of expected infections, 56% of expected hospitalizations, and 58% of expected deaths.

**Meaning:**

The US COVID-19 vaccination program was estimated to prevent substantial morbidity and mortality through direct protection of vaccinated individuals.

## Teaser

This modeling study of US adults vaccinated against COVID-19 estimates the number of infections, hospitalizations, and deaths prevented by the US COVID-19 vaccination program.

## Introduction

An estimated 16 million confirmed cases of COVID-19 and 310 000 COVID-19–associated deaths were reported in the US during the prevaccination period of the COVID-19 pandemic, defined as the period beginning with identification of the first US case of COVID-19 on January 19, 2020, through the beginning of the US vaccination program on December 12, 2020.^1,2^ In 2021, vaccination coverage of the adult and pediatric US populations increased after recommendations for phased allocation of initial supplies to long-term care facility residents, older adults, persons with high-risk medical conditions, health care personnel, and other frontline and essential workers.^3^ By September 30, 2021, approximately 67% of individuals 18 years or older and 83% of individuals 65 years or older had been fully vaccinated with 2 doses of the BNT162b2 (Pfizer-BioNTech) or mRNA-1273 (Moderna) or 1 dose of the JNJ-78436735 (Janssen) COVID-19 vaccine.^4^ Vaccine effectiveness (VE) studies^5,6,7,8,9,10,11^ have demonstrated that all 3 vaccines are highly effective against COVID-19–associated hospitalizations and deaths, despite the emergence of Delta and other SARS-CoV-2 variants.

Since December 12, 2020, an additional 27 million confirmed cases and 390 000 associated deaths had been reported as of September 30, 2021.^4^ However, the US burden of COVID-19 prevented among vaccinated persons, a key measure of the impact of the US vaccination program, has not been established. This study aims to estimate the numbers of infections, hospitalizations, and deaths prevented among persons who received a COVID-19 vaccine in the US.

## Methods

The Centers for Disease Control and Prevention determined that this modeling study, which involved no enrollment of human participants, did not require institutional review board approval or informed consent. This report follows the Consolidated Health Economic Evaluation Reporting Standards (CHEERS) reporting guideline where applicable.

### Combined Models for Extrapolating COVID-19 Burden

To estimate the burden of COVID-19 by age group (18-49, 50-64, and ≥65 years), month, and state, we obtained data on the estimated number of COVID-19 hospitalizations^12^ and used a multiplier model to extrapolate the number of SARS-CoV-2 infections (both asymptomatic and symptomatic infections) and COVID-19–associated deaths from hospitalizations. Multiplier models use probabilistic multipliers to account for sources of underascertainment (eg, probability of testing and probability of clinical outcomes being reported) to better estimate the true burden of a pathogen. This method has been used previously to estimate the burden of foodborne illness, the burden of influenza during the 2009 influenza pandemic, and the burden of COVID-19 in the US.^13,14,15^ In this study, we used age group–specific ratios of infections to hospitalizations to extrapolate numbers of SARS-CoV-2 infections.^13^ To estimate numbers of COVID-19 deaths, we used the age group–specific and month-specific proportions of hospitalizations that resulted in death estimated from COVID-NET data^16^ multiplied by the inverse proportion of COVID-19 coded deaths that occurred in hospitals among all COVID-19 coded deaths from the National Vital Statistics System (ie, to account for underreporting of COVID-19 deaths outside the hospital setting) (Table 1; eMethods, eTables 1-4, and eFigures 1 and 2 in the Supplement).^4,5,7,9,11,12,13,16,17,18,19,20,21,22,23,24,25,26,27,28,29,30,31^

**Table 1.** Parameter Values, Uncertainty Ranges, Assumptions, and Data Sources for COVID-19 Burden and Vaccination Models

Parameter	Value	Uncertainty range	Source
Estimated number of COVID-19 hospitalizations	Age, month, and state specific	Beta-PERT distributions based on medians and 10th and 90th percentiles of estimated hospitalizations^a^^,^^b^	Couture et al,^12^ 2021
Proportion of hospitalizations resulting in death	Age and month specific	Beta-PERT distributions based on point estimates and 95% CIs^c^	COVID-NET surveillance data^16^
Proportion of COVID-19 deaths occurring in the hospital	Age, month, and state specific	No uncertainty assumed^d^	National Center for Health Statistics provisional mortality data^17^
Ratio of infections to hospitalizations			
18-49 y of age	46.4	PERT (31.7-57.6)	Reese et al,^13^ 2020
50-64 y of age	15.2	PERT (10.8-22.1)	
≥65 y of age	6.0	PERT (4.8-7.7)	
Vaccine coverage	Age-, state- and month-specific counts of vaccine course completion	No uncertainty assumed^e^	COVID-19 vaccinations in the US, Centers for Disease Control and Prevention^4^
Vaccine effectiveness for BNT162b2 and mRNA-1273 (before Delta variant), %			
<65 y of age	90	PERT (85-95)	Andrejko et al,^18^ 2021; Amirthalingam et al,^19^ 2021; Bruxvoort et al,^20^ 2021; Butt et al,^21^ 2021; Chung et al,^22^ 2021; Iliaki et al,^23^ 2021; Mason et al,^24^ 2021; Moustsen-Helms et al,^25^ 2021; Nanduri et al,^11^ 2021; Pawlowski et al,^9^ 2021; Pilishvili et al,^26^ 2021; Thompson et al,^5^ 2021
≥65 y of age	75	PERT (65-85)	
Efficacy of BNT162b2 and mRNA-1273 (after Delta variant), %			
<65 y of age	75	PERT (60-80)	Chin et al,^27^ 2021; Barlow et al,^28^ 2021; Bruxvoort et al,^20^ 2021; Nanduri et al,^11^ 2021; Tartof et al,^7^ 2021
≥65 y of age	55	PERT (45-65)	
Efficacy of JNJ-78436735	75	PERT (65-85)	Corchado-Garcia et al,^29^ 2021; Iliaki et al,^23^ 2021; Lin et al,^30^ 2021; Polinski et al,^31^ 2021

Abbreviation: PERT, Program (or Project) Evaluation and Review Technique.

^a^ The beta-PERT distribution is a continuous probability distribution defined by a minimum value, a most likely value, and a maximum value.

^b^ Estimated numbers of COVID-19 hospitalizations are presented in eTable 1 in the Supplement.

^c^ Proportions of hospitalizations resulting in death are presented in eTable 3 in the Supplement.

^d^ Proportions of COVID-19 deaths occurring in the hospital are presented in eTable 4 in the Supplement.

^e^ Vaccine coverage data are presented in eTable 5 in the Supplement.

### Vaccine Coverage Data and VE Estimates

We obtained counts of persons who completed a vaccine series (ie, 2 doses for BNT162b2 and mRNA-1273 or 1 dose for JNJ-78436735) by age group, month, state, and vaccine product, as reported to the CDC by September 30, 2021. These vaccination coverage estimates were obtained using vaccine administration data reported to the CDC from state and jurisdiction immunization information systems and the Vaccine Administration Management System. Because these were monthly aggregate counts of individuals who completed a vaccine series, these counts consisted of individuals who were at least 14 days past completion of their vaccine series (ie, fully vaccinated) and those who were less than 14 days past completion. We used a simplifying assumption that all those who completed a vaccine series were fully vaccinated; however, we ran our models over a range of VE estimates as described below (Table 1). Our analysis excludes the impact of partial vaccination among persons receiving a single dose of BNT162b2 or mRNA-1273 and excludes the impact of additional or booster doses.

We parameterized product-specific VE estimates using data from a literature review compiled by the International Vaccine Access Center and the World Health Organization.^32^ We restricted estimates of VE against documented infection among fully vaccinated persons to peer-reviewed studies conducted after authorization in the US or Western Europe.^7,11,18,21,22,23,25,28,29^ On the basis of this subset of studies, we used a single VE estimate applied to all ages for JNJ-78436735 vaccine; we stratified VE estimates for BNT162b2 and mRNA-1273 by those younger than 65 years and those 65 years or older and by pre-Delta (December 1, 2020, to May 31, 2021) and post-Delta (June 1 to September 30, 2021) periods (Table 1).^33^ Because of the model’s structure, when vaccination prevents infection, individuals are not subsequently at risk for hospitalization or death. As such, the model does not account for any additional reductions in risk from vaccination (ie, VE against hospitalization and deaths is effectively equivalent to VE against infection, which is a conservative, simplifying assumption) (eFigure 3 in the Supplement). However, we evaluated the effects of our framework by specifying a vaccination model with the pre-Delta VE for BNT162b2 and mRNA-1273 fixed over time (eTable 6 in the Supplement). We also ran our model across a range of VE values to incorporate uncertainty into our estimates of the burden of COVID-19 prevented by vaccination (Table 1).

### Statistical Analysis

We adapted a compartmental model developed for influenza to estimate the burden of COVID-19–associated disease prevented among vaccinated persons.^34^ Using data on observed vaccine coverage, estimates of VE, and estimates of infections, the model estimates the numbers of persons who become infected with SARS-CoV-2, persons who are vaccinated and presumed immune to SARS-CoV-2 infection, and persons who remain susceptible to infection by age group, month, and 50 US states (excluding the District of Columbia) (eFigure 4 in the Supplement).

We estimated age-specific risks of infection, hospitalizations, and deaths for each month and state by dividing the monthly numbers of each outcome by the number of individuals who were susceptible by the end of the previous month. To estimate the expected burden of COVID-19 in the absence of vaccination, we multiplied the estimated risks of infection, hospitalizations, and deaths for each age group, month, and state by the estimated numbers of susceptible individuals in each equivalent age group, month, and state in the absence of vaccination. The prevented burden of COVID-19 was calculated as the difference between expected outcomes in the absence of vaccination and the observed burden with vaccination. We summed the estimates of the prevented COVID-19 burden across all states and months to generate national-level estimates and summed the prevented burden to generate estimates by US Department of Health and Human Services (HHS) regions.^35^ We calculated the age group– and month-specific numbers of COVID-19 outcomes prevented per 100 000 population by dividing the estimated number of COVID-19 outcomes prevented by age group–specific US Census population size estimates.^36^ The percentages of COVID-19 outcomes prevented were calculated by dividing burden estimates under current levels of vaccination by expected outcomes in the absence of vaccination (eMethods in the Supplement). We ran 5000 Monte Carlo simulations of the vaccination model using a range of values for key parameters (including VE, infections to hospitalizations ratio, and deaths to hospitalizations ratio) (Table 1; eMethods, eTables 3-5 in the Supplement) to generate medians and 95% uncertainty intervals (UIs), defined as the 2.5th and 97.5th percentiles, for the numbers and percentages of infections, hospitalizations, and deaths prevented by COVID-19 vaccination. All analyses were conducted in R, version 4.0.5 (R Foundation for Statistical Computing).^37^

## Results

### Estimates of COVID-19 Clinical Outcomes

Couture et al^12^ previously estimated that approximately 3.36 million (95% UI, 3.2 million to 3.4 million) COVID-19–associated hospitalizations occurred between December 1, 2020, and September 30, 2021^2^ (eMethods in the Supplement). Using these hospitalization estimates as input data in our multiplier model, we estimated that 69 million (95% UI, 59 million to 78 million) SARS-CoV-2 infections and 431 000 (95% UI, 357 000-491 000) COVID-19–associated deaths occurred in the US during the same time frame. Individuals 18 to 49 years of age had the highest cumulative number of infections per 100 000 population (29 000 [95% UI, 23 000-35 000]), whereas individuals 65 years or older had the highest cumulative number of hospitalizations (2740 [95% UI, 2670-2800]) and deaths (560 [95% UI, 430-660]) per 100 000 population. The prevalent number of clinical outcomes across all age groups had a U-shaped distribution over time, with the lowest prevalence of infections, hospitalizations, and deaths occurring from June 1 to June 30, 2021, and higher prevalences occurring from December 1, 2020, to January 31, 2021, and August 1 to September 30, 2021 (eFigure 1 in the Supplement).

### Estimates of COVID-19 Clinical Outcomes Prevented by Vaccination

The US COVID-19 vaccination program was estimated to prevent 27 million (95% UI, 22 million to 34 million) infections, 1.6 million (95% UI, 1.4 million to 1.8 million) hospitalizations, and 235 000 (95% UI, 175 000-305 000) deaths among vaccinated persons 18 years or older from December 1, 2020, to September 30, 2021 (Table 2). Most of the estimated hospitalizations and deaths prevented were among those 65 years or older (759 000 hospitalizations; 95% UI, 622 000-946 000; 154 000 deaths, 95% UI, 105 000-214 000) and those 50 to 64 years of age (525 000 hospitalizations; 95% UI, 454 000-646 000; 66 000 deaths; 95% UI, 40 000-94 000).

**Table 2.** Estimates of SARS-CoV-2 Infections and COVID-19–Associated Hospitalizations and Deaths Prevented by Direct Effects of COVID-19 Vaccination in the US, December 1, 2020, to September 30, 2021

Age group, y	No. prevented (95% UI)		
	Infections	Hospitalizations	Deaths
18-49	13 700 000 (10 000 000-18 600 000)	301 000 (260 000-367 000)	14 000 (7500-20 000)
50-64	8 100 000 (5 800 000-12 000 000)	525 000 (454 000-646 000)	66 000 (40 000-94 000)
≥65	4 600 000 (3 500 000-6 100 000)	759 000 (622 000-946 000)	154 000 (105 000-214 000)
Total (≥18 y)^a^	26 700 000 (22 000 000-34 000 000)	1 600 000 (1 400 000-1 800 000)	235 000 (175 000-305 000)

Abbreviation: UI, uncertainty interval.

^a^ Point estimates for the total outcomes prevented will not necessarily equal to the sum of the age group–specific point estimates. They represent the median total outcomes prevented from 5000 simulations of the model.

Vaccination was estimated to prevent 30% (95% UI, 27%-33%) of all expected infections, 33% (95% UI, 30%-36%) of all expected hospitalizations, and 34% (95% UI, 28%-40%) of all expected deaths in adults 18 years or older from December 1, 2020, to September 30, 2021. As vaccine coverage increased over time, the estimated percentage of expected infections, hospitalizations, and deaths prevented increased. In September, vaccination was estimated to prevent 52% (95% UI, 45%-62%) of expected infections, 56% (95% UI, 52%-62%) of expected hospitalizations, and 58% (95% UI, 53%-63%) of expected deaths in adults 18 years or older. The populations 50 to 64 years of age and 65 years or older have had the highest estimated numbers of hospitalizations and deaths prevented per 100 000 population over time (Figure; eFigure 1 in the Supplement). Among the population 50 to 64 years of age, the estimated number of prevented hospitalizations per 100 000 population ranged from 10 (95% UI, 9-11) in March 2021 to 327 (95% UI, 233-478) in September 2021, and estimated deaths prevented per 100 000 population ranged from 0.8 (95% UI, 0.3-1.2) in March 2021 to 51 (95% UI, 16-88) in September 2021. The estimated number of hospitalizations prevented per 100 000 population among those 65 years or older ranged from 34 (95% UI, 30-40) in March 2021 to 484 (95% UI, 389-612) in September 2021, and the estimated number of deaths prevented per 100 000 population ranged from 6 (95% UI, 2-8) in March 2021 to 96 (95% UI, 36-153) in August 2021.

**Figure.**
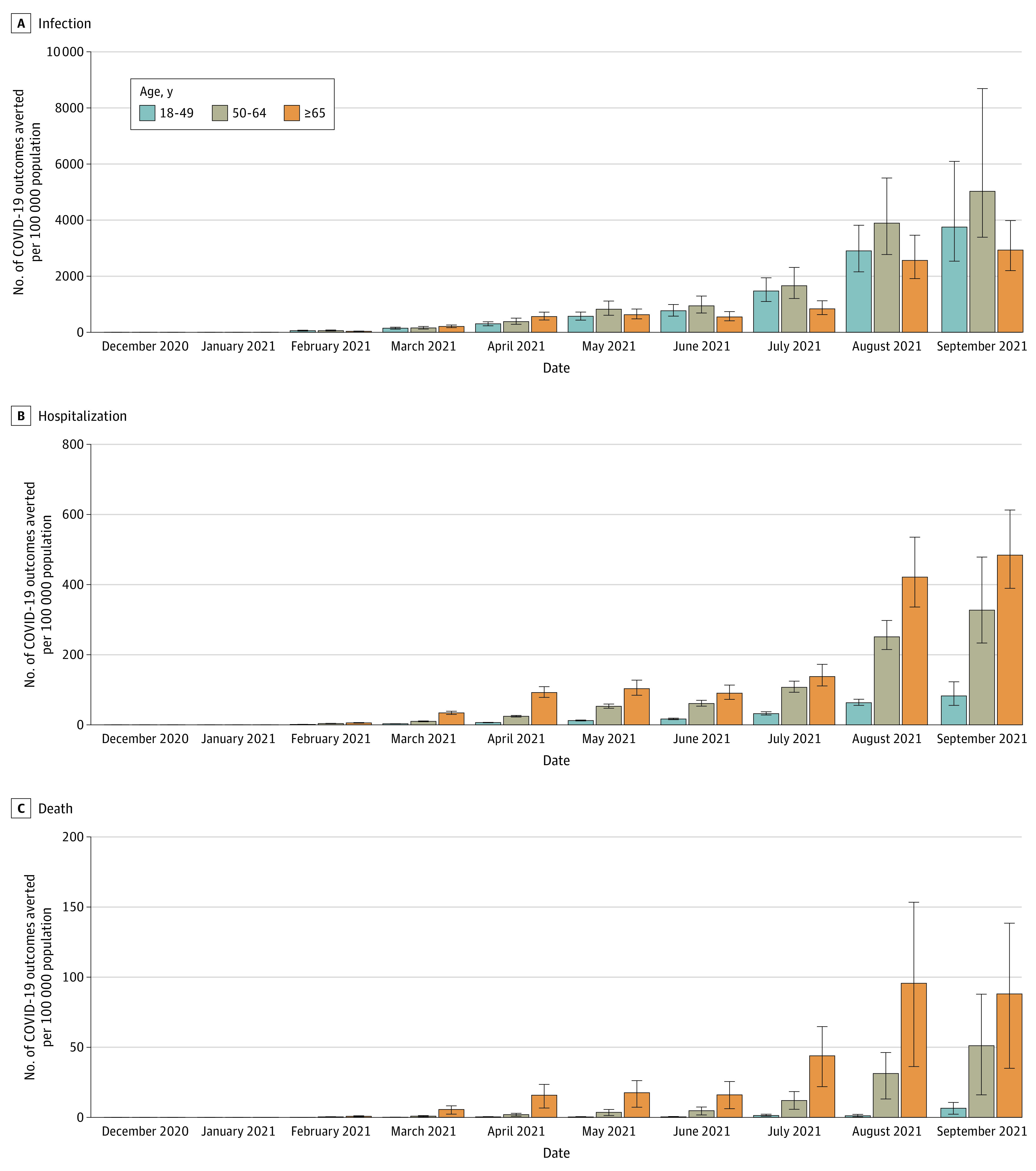
Median Estimated Monthly Number of Expected SARS-CoV-2 Infections and COVID-19–Associated Hospitalizations and Deaths Prevented by Vaccination per 100 000 Population by Age Group From December 1, 2020 to September 30, 2021, in the US Error bars indicate 95% uncertainty intervals.

The estimated percentages of infections, hospitalizations, and deaths prevented by vaccination varied across HHS regions (Table 3) because of regional differences in vaccine coverage over time (eTable 5 in the Supplement). From December 1, 2020, to September 30, 2021, HHS region 10 had the highest estimated percentage of expected infections (36%; 95% UI, 34%-39%), hospitalizations (40%; 95% UI, 38%-44%), and deaths (41%; 95% UI, 36%-47%) prevented among adults 18 years or older; HHS region 3 had the lowest estimated percentage of expected infections (27%; 95% UI, 25%-30%), hospitalizations (28%; 95% UI, 26%-31%), and deaths (29%; 95% UI, 23%-35%) prevented among adults 18 years or older. From September 1, 2021, to September 30, 2021, HHS region 2 had the highest estimated percentage of expected infections (63%; 95% UI, 60%-65%), hospitalizations (64%; 95% UI, 61%-67%), and deaths (65%; 95% UI, 61%-68%) prevented among adults 18 years or older, whereas HHS region 4 had the lowest estimated percentage of expected infections (44%; 95% UI, 42%-47%), hospitalizations (51%; 95% UI, 48%-54%), and deaths (52%; 95% UI, 49%-57%) prevented among adults 18 years or older.

**Table 3.** Estimates of the Percentage of Expected SARS-CoV-2 Infections and COVID-19 Hospitalizations and Deaths Prevented by Direct Effects of COVID-19 Vaccination by HHS Region, December 1, 2020, to September 30, 2021, and September 1 to September 30, 2021

HHS region^a^	Estimated expected outcomes prevented, % (95% UI)		
	Infections	Hospitalizations	Deaths
**December 1, 2020, to September 30, 2021**			
1	31 (29-34)	32 (29-34)	32 (27-38)
2	30 (28-32)	29 (27-31)	27 (22-33)
3	27 (25-30)	28 (26-31)	29 (23-35)
4	27 (25-29)	33 (30-36)	36 (31-42)
5	30 (28-32)	33 (31-36)	33 (27-39)
6	31 (21-45)	35 (28-44)	38 (30-47)
7	29 (27-31)	32 (30-35)	34 (29-40)
8	32 (30-34)	35 (33-38)	35 (30-41)
9	31 (29-33)	31 (29-33)	32 (27-37)
10	36 (34-39)	40 (38-44)	41 (36-47)
**September 1-30, 2021**			
1	61 (59-63)	63 (60-65)	64 (60-67)
2	63 (60-65)	64 (61-67)	65 (61-68)
3	57 (54-60)	60 (57-63)	61 (57-64)
4	44 (42-47)	51 (48-54)	52 (49-57)
5	54 (51-56)	59 (56-62)	60 (57-64)
6	55 (24-87)	58 (41-81)	59 (44-78)
7	48 (45-50)	53 (50-57)	56 (52-60)
8	54 (51-56)	57 (54-59)	58 (54-61)
9	54 (52-56)	56 (54-58)	57 (54-60)
10	54 (52-57)	58 (55-61)	59 (56-63)

Abbreviations: HHS, US Department of Health and Human Services; UI, uncertainty interval.

^a^ HHS region 1 includes Connecticut, Maine, Massachusetts, New Hampshire, Rhode Island, Vermont; region 2, New Jersey, New York; region 3, Delaware, Maryland, Pennsylvania, Virginia, and West Virginia; region 4, Alabama, Florida, Georgia, Kentucky, Mississippi, North Carolina, South Carolina, and Tennessee; region 5, Illinois, Indiana, Michigan, Minnesota, Ohio, and Wisconsin; region 6, Arkansas, Louisiana, New Mexico, Oklahoma, and Texas; region 7, Iowa, Kansas, Missouri, and Nebraska; region 8, Colorado, Montana, North Dakota, South Dakota, Utah, and Wyoming; region 9, Arizona, California, Hawaii, and Nevada; and region 10, Alaska, Idaho, Oregon, and Washington.

## Discussion

The US COVID-19 vaccination program was estimated to prevent approximately 27 million infections, 1.6 million hospitalizations, and 235 000 deaths from December 1, 2020, to September 30, 2021, among vaccinated adults 18 years or older. In these first 10 months of the program, 2 major surges in the pandemic occurred. The program began during the peak of the largest surge in the winter of 2020 and continued as the SARS-CoV-2 Delta variant rose to predominance in June 2021, likely causing a decrease in VE against infection.^38^ As vaccination coverage increased in 2021, the COVID-19 vaccination program increasingly functioned as intended, with the highest impact averting severe disease among older adults. These age groups have not only the highest rates of hospitalizations and deaths but also the highest vaccine coverage. We also identified regional differences in vaccine impact; from September 1 to September 30, 2021, the northeastern US (HHS regions 1 and 2) had the highest estimated COVID-19 burden prevented because those regions had high COVID-19 vaccination coverage rates.^4^ Some of the variation in regional-level impact may be explained in part by differences in age. For example, deaths are predominantly experienced by older age groups; thus, regions that have a higher proportion of older individuals may have a higher percentage of deaths averted in the total population despite having lower vaccine coverage compared with other HHS regions with younger age distributions.

This study is among the first, to our knowledge, to present estimates of age group–specific impacts of vaccination among vaccinated persons in the US over time and by HHS region. Our estimates combined a multiplier model to extrapolate COVID-19 burden and a simple compartmental model of vaccination using population vaccine coverage data and VE estimates from the published literature. Using different modeling approaches, several other published studies have also estimated the impact of COVID-19 vaccination in the US. Gupta et al^39^ estimated that US vaccination prevented approximately 139 000 deaths (95% CI, 13 110-265 675) in individuals 16 years or older by May 9, 2021. Our modeling approach estimated approximately 27 000 (95% UI, 14 000-38 000) deaths prevented by vaccination through the end of May 2021. These differences are likely attributable to distinct analytic methods, underlying assumptions, and the application of different data sources. Unlike Gupta et al,^39^ we estimated deaths prevented by age group using age-specific vaccine coverage data for persons who completed a vaccine series and accounting for VE by product and age group. Our estimates, particularly in the early part of 2021, are relatively conservative because most vaccinated individuals during this period were older adults (ie, older adults were prioritized for vaccination early compared with younger adults); we also included lower VE estimates for those 65 years or older. Samson et al^40^ used a regression model approach similar to that of Gupta et al^39^ to estimate that approximately 21% of hospitalizations were prevented among Medicare fee-for-service beneficiaries between January and May 2021. Our modeling approach produced a lower estimate of approximately 14% of hospitalizations in adults 18 years or older prevented for the same period. These differences may be attributable to demographic differences between Medicare beneficiaries and the overall US population. Assuming that most Medicare beneficiaries in 2021 were 65 years or older (86%), a higher proportion of Medicare beneficiaries were likely vaccinated earlier, which would result in higher estimates of vaccine impact.^41^ Moghadas et al^42^ estimated that 26 million cases, 1.2 million hospitalizations, and 279 000 deaths were prevented by vaccination between December 12, 2020, and June 28, 2021, compared with our estimates of 5.1 million infections, 332 000 hospitalizations, and 39 000 deaths prevented in the same period. Mostly notably, the approach used by Moghadas et al^42^ accounts for direct and indirect benefits (ie, protection provided to unvaccinated persons through reduced transmission) of vaccination.

### Limitations

This study has limitations. Our estimates of infections, hospitalizations, and deaths prevented focus specifically on the benefit of vaccination among vaccinated persons. The model does not consider reductions in infections, hospitalizations, or deaths in unvaccinated people because of vaccine-induced reductions in transmission. Therefore, our estimates represent a portion of the total burden of COVID-19 prevented by vaccination in the US. Furthermore, our model does not account for any marginal benefits among those who are partially vaccinated or acquired immunity from previous infections, which may lead to underestimation of the burden of COVID-19 prevented among vaccinated persons. We did not account for waning immunity in the model but incorporated a reduction in VE during the period when the Delta variant was dominant. In addition, we assume that the risks of hospitalization and death are the same between the population with observed levels of vaccination and the counterfactual population without vaccination. If vaccination further reduced the population-level risk of severe disease, such as hospitalizations and deaths, this reduction would lead to underestimation of the expected burden in a population without vaccination and subsequently to underestimation of the burden of COVID-19 prevented by vaccination. Additionally, our estimated inputs for the number of infections, hospitalizations, and deaths were derived from previously published COVID-19 burden models, which also have recognized limitations.^12,13^ Our input data are estimates of hospitalizations with positive test results for SARS-CoV-2 infections. Currently, COVID-NET surveillance data do not indicate whether patients were hospitalized for complications caused by SARS-CoV-2 infection. In addition, these hospitalization estimates were calculated assuming that COVID-NET captures all patients who were tested for SARS-CoV-2 and had a positive result. Although the estimates of hospitalizations were adjusted for testing practices, these may be underestimated if not all hospitalized patients with confirmed SARS-CoV-2 were reported. Further limitations for these estimates of hospitalizations are cited in the study by Couture et al.^12^

## Conclusions

This study’s findings indicate that COVID-19 vaccination in the US has provided substantial protection against infections, hospitalizations, and deaths among those who have been vaccinated. Although the estimates are conservative, they demonstrate the direct benefits of the US COVID-19 vaccination program. Future models and analyses could estimate the impact of vaccination among individuals younger than 18 years, the benefits of partial vaccination, indirect benefits of vaccination on disease transmission, and the impact of additional primary or booster doses. Vaccination is an effective public health intervention with demonstrable impact, which will be critical in combination with nonpharmaceutical interventions to mitigate the COVID-19 pandemic.

## Notes

Supplement. **eMethods.** Supplemental Methods **eFigure 1.** Estimated Prevalent Numbers of SARS-Cov-2 Infections and COVID-19-Associated Hospitalizations and Deaths per 100,000 Population by Month and Age Group **eFigure 2.** Flow Diagram of the Methods to Estimate SARS-Cov-2 Infections and COVID-19-Associated Hospitalizations and Deaths Prevented by Vaccination **eFigure 3.** Distribution of Outcomes in Model Framework **eFigure 4.** Burden Prevented Model Schematic **eTable 1.** Estimates (95% Uncertainty Interval) of Observed Number of COVID-19 Hospitalizations During December 1, 2020 - September 30, 2021 **eTable 2.** Percent of Place of Death Data Suppressed **eTable 3.** Median Proportion (Range) of Hospitalizations Resulting in Death by Age Group and Month in the U.S. **eTable 4.** Estimated Proportion of COVID-19-Associated Deaths Occurring in Hospital Settings Out of All Places of Death **eTable 5.** Percent of ≥18 Year Olds That Had Completed Vaccine Series by HHS Region and Month **eTable 6.** Estimates (95% Uncertainty Interval) of SARS-Cov-2 Infections and COVID-19-Associated Hospitalizations and Deaths Directly Prevented by COVID-19 Vaccination in the U.S During December 1, 2020 – September 30, 2021, Assuming Fixed, Pre-Delta VE Over Time
eReferences
