# Supplementary material for: Estimated Number of COVID-19 Infections, Hospitalizations, and Deaths Prevented Among Vaccinated Persons in the US, December 2020 to September 2021
Source: JAMA Netw Open. 2022 Jul 6;5(7):e2220385. doi: 10.1001/jamanetworkopen.2022.20385 (PMC9260489; doi:10.1001/jamanetworkopen.2022.20385)
Supplement: Supplement. — eMethods. Supplemental Methods eFigure 1. Estimated Prevalent Numbers of SARS-Cov-2 Infections and COVID-19-Associated Hospitalizations and Deaths per 100,000 Population by Month and Age Group eFigure 2. Flow Diagram of the Methods to Estimate SARS-Cov-2 Infections and COVID-19-Associated Hospitalizations and Deaths Prevented by Vaccination eFigure 3. Distribution of Outcomes in Model Framework eFigure 4. Burden Prevented Model Schematic eTable 1. Estimates (95% Uncertainty Interval) of Observed Number of COVID-19 Hospitalizations During December 1, 2020 - September 30, 2021 eTable 2. Percent of Place of Death Data Suppressed eTable 3. Median Proportion (Range) of Hospitalizations Resulting in Death by Age Group and Month in the U.S. eTable 4. Estimated Proportion of COVID-19-Associated Deaths Occurring in Hospital Settings Out of All Places of Death eTable 5. Percent of ≥18 Year Olds That Had Completed Vaccine Series by HHS Region and Month eTable 6. Estimates (95% Uncertainty Interval) of SARS-Cov-2 Infections and COVID-19-Associated Hospitalizations and Deaths Directly Prevented by COVID-19 Vaccination in the U.S During December 1, 2020 – September 30, 2021, Assuming Fixed, Pre-Delta VE Over Time eReferences [file jamanetwopen-e2220385-s001.pdf]

Supplementary Online Content

Steele MK, Couture A, Reed C, et al. Estimated number of COVID-19 infections, hospitalizations, and deaths prevented among vaccinated persons in the US, December 2020 to September 2021. *JAMA Netw Open*. 2022;5(7):e2220385. doi:10.1001/jamanetworkopen.2022.20385

eMethods. Supplemental Methods

eFigure 1. Estimated Prevalent Numbers of SARS-Cov-2 Infections and COVID-19-Associated Hospitalizations and Deaths per 100,000 Population by Month and Age Group

eFigure 2. Flow Diagram of the Methods to Estimate SARS-Cov-2 Infections and COVID-19-Associated Hospitalizations and Deaths Prevented by Vaccination

eFigure 3. Distribution of Outcomes in Model Framework

eFigure 4. Burden Prevented Model Schematic

eTable 1. Estimates (95% Uncertainty Interval) of Observed Number of COVID-19 Hospitalizations During December 1, 2020 - September 30, 2021

eTable 2. Percent of Place of Death Data Suppressed

eTable 3. Median Proportion (Range) of Hospitalizations Resulting in Death by Age Group and Month in the U.S.

eTable 4. Estimated Proportion of COVID-19-Associated Deaths Occurring in Hospital Settings Out of All Places of Death

eTable 5. Percent of ≥18 Year Olds That Had Completed Vaccine Series by HHS Region and Month

eTable 6. Estimates (95% Uncertainty Interval) of SARS-Cov-2 Infections and COVID-19-Associated Hospitalizations and Deaths Directly Prevented by COVID-19 Vaccination in the U.S During December 1, 2020 – September 30, 2021, Assuming Fixed, Pre-Delta VE Over Time

eReferences

This supplementary material has been provided by the authors to give readers additional information about their work.

eMethods. Supplemental Methods

Input Data:

Hospitalization Data

Our model input data for the numbers of COVID-19-association hospitalizations stratified by age group, month, and state were previously estimated by Couture et al.[1] Briefly, Couture et al. obtained data on age group (0-17, 18-49, 50-64, 65-74, 75-84, and ≥85 years), month, and state-specific hospitalization counts collected by the Coronavirus Disease 2019 (COVID-19)-Associated Hospitalization Surveillance Network (COVID-NET).[1] (eTable 1) COVID-NET is a population-based surveillance network of 250 acute-care hospitals in 14 states (California, Colorado, Connecticut, Georgia, Iowa, Maryland, Michigan, Minnesota, New Mexico, New York, Ohio, Oregon, Tennessee, and Utah) that covers about 10% of the U.S. population. A COVID-19 hospitalization was defined as a patient hospitalized within 14 days of a positive result from a SARS-CoV-2 test ordered by a healthcare professional.

It is unlikely that all true COVID-19-associated hospitalized patients are captured in surveillance as 1) not all patients may be tested for SARS-CoV-2 and 2) some patients may have false negative tests based on assay sensitivity. To account for these factors that contribute to underreporting of COVID-19-associated hospitalizations, Couture et al. adjusted COVID-NET hospitalization counts by age group and month-specific probabilities of SARS-CoV-2 testing calculated from IBM Watson Health Explorys electronic health records, and by SARS-CoV-2 assay sensitivity. After adjustment, Couture et al. used six Bayesian hierarchical models (one for each age group) stratified by state and month to extrapolate hospitalization counts from COVID-NET sites to states not captured by COVID-NET surveillance. Model covariates varied for the different age group models, however covariates considered for inclusion across models were: SARS-CoV-2 RT-PCR test positivity (stratified by age group, month and state), percent of all-cause deaths that were coded as COVID-19-associated (stratified by age group, month and state), hospital capacity metrics (percent COVID patients out of all inpatients and percent of ICU beds occupied by COVID patients stratified by month and state), race/ethnicity percentages (percent American Indian, Black or racial minority stratified by state and age) and chronic conditions percentages (percent any chronic, obesity, hear disease COPD, diabetes, chronic kidney disease and asthma stratified by state). These models were run for 20,000 Markov chain Monte Carlo (MCMC) iterations.

© 2022 Steele MK et al. *JAMA Network Open.*

Provisional COVID-19-Associated Place of Death Data

Death data were reported to the National Vital Statistics System (NVSS) of the National Center for Health Statistics (NCHS) from death certificates, which contained decedents' demographic information, date and place of death, and cause (or causes if multiple) of death.[2] We obtained provisional COVID-19 place of death data from NCHS, stratified by age group (18-49, 50-64, ≥65), month and state and estimated the proportion of deaths that occurred in inpatient settings out of all places of death. Not all states reported data for each age group and month; if no observed counts of deaths were reported for a given state, month and age group, (i.e., all values across all places of death were 0), we used the estimated proportion of deaths that occurred in inpatient settings out of all places of death from the corresponding HHS Region. Additionally, place of death data were suppressed if the number of COVID-19 deaths by age group, month and state was greater than 0 but fewer than 10 for a given state, month, and age group. Generally, for individuals aged 18-49 years suppression occurred more often for inpatient place of death than non-inpatient place of death, whereas in individuals 50-64 and ≥65 years old suppression occurred more often for non-inpatient place of death. (eTable 2) We replaced values for suppressed cells by randomly assigning values of 1 through 9, then calculated the proportion of deaths in hospital settings out of all places of death.

Multiplier Model to Estimate of COVID-19 Clinical Outcomes:

We obtained data on the number of COVID-19-associated hospitalizations by age group, month, and state estimated by a Bayesian hierarchical model.[1] Briefly, We drew 5,000 samples randomly between the 10th and 90th percentiles of 20,000 samples from a posterior distribution of estimated numbers of hospitalizations for each age group, month, and state; these samples were used as inputs for multiplier and vaccination models. (eTable 1) We chose to sample between the 10th and 90th percentiles of the posterior distribution of hospitalizations estimates as this falls in line with the results presented in Couture et al. and from our preliminary analyses we found that inclusion of hospitalizations estimates outside those percentiles (i.e. samples >90th percentile) resulted in biologically implausible estimates of burden averted by vaccination. To estimate the number of infections and deaths, we ran 5,000 Monte Carlo iterations of multiplier models for each age group, month and state. We estimated the observed number of infections (*Io*) by age group (*a*), month (*m*), and state (*s*) ($Io_{a,m,s}$) as follows:

$$Io_{a,m,s} = IH_a * Ho_{a,m,s}$$

© 2022 Steele MK et al. *JAMA Network Open.*

Where $Ho_{a,m,s}$ represents the observed numbers of hospitalizations for each age group, month, and state (estimated from the Bayesian hierarchical model described above) and $IH_{a,}$ represents the age group-specific ratio of infections to hospitalizations estimated from a multiplier model developed by Reese *et al*.[3] We sampled 5,000 values of the age group-specific ratio of infections to hospitalizations from a beta PERT distribution (see Table 1 of main text).

We estimated the observed number of deaths by age group, month, and state ($Do_{a,m,s}$) as follows:

$$Do_{a,m,s} = DH_{a,m} * \frac{1}{P(D)_{a,m,s}} * Ho_{a,m,s}$$

Where $DH_{a,m,}$ represents the age group- and month- specific ratio of deaths to hospitalizations estimated from COVID-NET surveillance data and $P(D)_{a,m,s}$ represents the age group, month and state specific proportion of deaths in hospital settings out of all places of death estimated from NVSS provisional COVID-19 place of death data (as described above). We sampled 5,000 values of the age group- and month-specific ratio of deaths to hospitalizations from a beta PERT distribution (eTable 3) and assumed fixed values for the age group, month and state specific proportion of deaths in hospital settings out of all places of death. (eTable 4)

Burden Prevented Model:

Our vaccination model is adapted from a previously published model of vaccination for seasonal influenza.[4] eFigure 2 presents the compartmental model framework to estimate the number of SARS-CoV-2 infections and COVID-19-associated hospitalizations and deaths prevented by vaccination in the United States. We used an age- and state-stratified compartmental model to estimate changes in the susceptible population by month under observed levels of vaccination. (eFigure 2A) The population was separated into 3 compartments based on vaccination and infection status. All population members were susceptible (S) at the beginning of the modeling timeframe, May 1, 2020. While this assumption did not account for pre-existing immunity from COVID-19 prior to May 1, 2020, a previous evaluation showed that accounting for persons with pre-existing immunity at the start of the model timeframe had minimal influence on the estimated burden prevented if the probability of vaccination did not differ by whether individuals had pre-existing immunity.[4] For each month (m), individuals of a given age group (a) and state (s) were removed from the susceptible ($S_{a,m,s}$) compartment by the number of observed infections in a given age group and

© 2022 Steele MK et al. *JAMA Network Open.*

state ($Io_{a,m,s}$) or by the observed age group-, state-, and vaccine product-specific coverage ($vc_{a,m,p,s}$). We assumed vaccination was take-type (i.e., individuals became fully immune or remained fully susceptible), therefore a proportion of individuals (determined by age group- and vaccine product-specific effectiveness during either the pre-Delta period from December 1, 2020 – May 31, 2021 or post-Delta period from June 30, 2021-September 30, 2021, $ve_{a,d,p}$) became vaccine protected ($V_{a,m,p,s}$) while the remaining proportion stayed susceptible (i.e., $S_{a,m,s}$ comprises individuals who were susceptible and unvaccinated and vaccinated but not protected). Using the methodology from Tokars *et al.,* we assume that susceptible and non-susceptible individuals had equal probability of being vaccinated.[4] We estimated the non-case population (i.e., susceptible and non-susceptible individuals) as follows:

$$if\ m = 1, \quad NC_{a,m,s} = Population - Io_{a,m,s}$$

$$else, \quad NC_{a,m,s} = NC_{a,m-1,s} - Io_{a,m,s}$$

The starting value for the non-case population (i.e., $m=1$) for a given age group and state ($NC_{a,m,s}$) was equal to the age group and state-specific population size minus the observed age group and state-specific infections in the first month ($Io_{a,m,s}$). After the first month, the non-case population was calculated as the observed age group and state-specific infections in a given month subtracted from the age group and state-specific non-case population from the previous month ($NC_{a,m-1,s}$). Based on these estimates of the non-case population, we calculated the number effectively vaccinated (i.e., those who gain immune protection from vaccination) in a given month as:

$$EV_{a,m,s} = NC_{a,m-1,s} \sum_{p}^{1} vc_{a,m,p,s} ve_{a,d,p}$$

Where $vc_{a,m,p,s}$ represents the month and state-specific proportion of age group a vaccinated by product p, and $ve_{a,d,p}$ the age group-specific vaccine effectiveness of vaccine product p in time period d (i.e, pre-Delta vaccine effectiveness defined as December 1, 2020 – May 31, 2021 and post-Delta effectiveness defined as June 1, 2021 – September 30, 2021). We sampled 5,000 values for each age group-, vaccine product-, and time period-specific vaccine effectiveness from a beta PERT distribution (see Table 1 of main text), and assumed fixed values for the age group-, month-, vaccine product-, and state-specific vaccine coverage (eTable 5).

© 2022 Steele MK et al. *JAMA Network Open.*

In this model, infections were used to estimate changes in the susceptible population. If vaccination prevented an infection, individuals did not progress in susceptibility to hospitalization or death. As such, the numbers of hospitalizations and deaths were subsets of the number of infections (eFigure 3). Hospitalizations were comprised of fatal and non-fatal hospitalizations. Deaths were comprised of those who were previously hospitalized and those never hospitalized. For each age group, state, and month we calculated the risks of infections ($P(I)_{a,m,s}$), hospitalizations ($P(H)_{a,m,s}$), and deaths ($P(D)_{a,m,s}$) as follows:

$$P(I)_{a,m,s} = \frac{Io_{a,m,s}}{S_{a,m-1,s}}$$

$$P(H)_{a,m,s} = \frac{Ho_{a,m,s}}{S_{a,m-1,s}}$$

$$P(D)_{a,m,s} = \frac{Do_{a,m,s}}{S_{a,m-1,s}}$$

Where $Io_{a,m,s}$, $Ho_{a,m,s}$, and $Do_{a,m,s}$ are the estimated inputs of the observed number of infections, hospitalizations and deaths, respectively, by age group, month, and state given the observed levels of vaccine coverage. $S_{a,m-1,s}$ represent the age- and state-specific counts of susceptible individuals from the previous month.

To estimate the expected burden of COVID-19 in the absence of vaccination, we used a second age- and state-stratified compartmental model where individuals are only removed from the susceptible compartment ($S_{a,m,s}$) by the estimated risk of infection ($P(I)_{a,m,s}$) (eFigure 2B). Age-, month- and state-specific counts of expected of infections, hospitalizations and deaths in the absence of vaccination were calculated as follows:

$$Ie_{a,m,s} = P(I)_{a,m,s} * S_{a,m,s}$$

$$He_{a,m,s} = P(H)_{a,m,s} * S_{a,m,s}$$

$$De_{a,m,s} = P(D)_{a,m,s} * S_{a,m,s}$$

© 2022 Steele MK et al. *JAMA Network Open.*

eFigure 1. Estimated prevalent numbers of SARS-CoV-2 infections and COVID-19-associated hospitalizations and deaths per 100,000 population by month and age group.

Colored bars represent median estimates and black lines represent 95% uncertainty intervals.

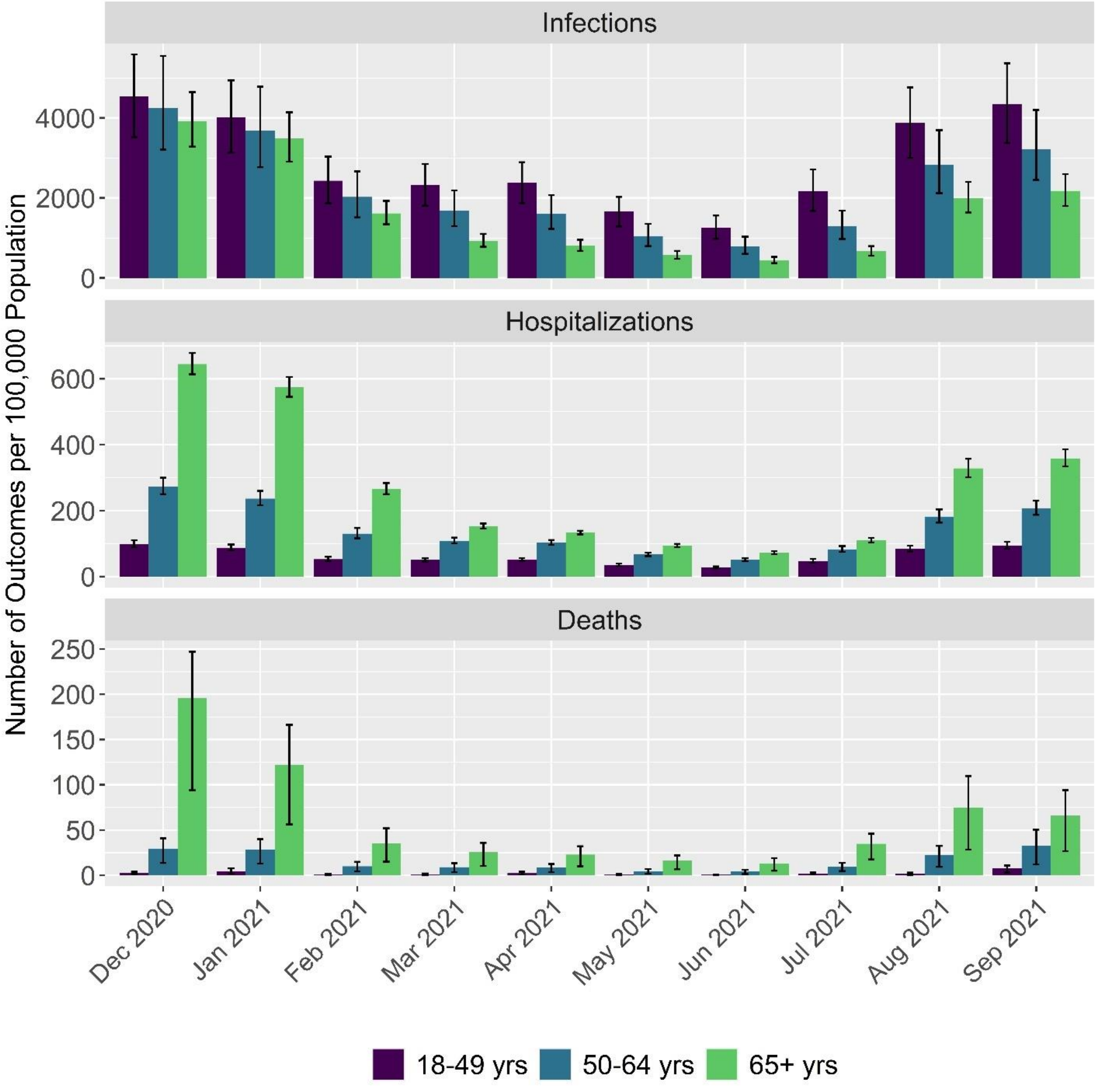

© 2022 Steele MK et al. *JAMA Network Open.*

eFigure 2. Flow diagram of the methods to estimate SARS-CoV-2 infections and COVID-19-associated hospitalizations and deaths prevented by vaccination.

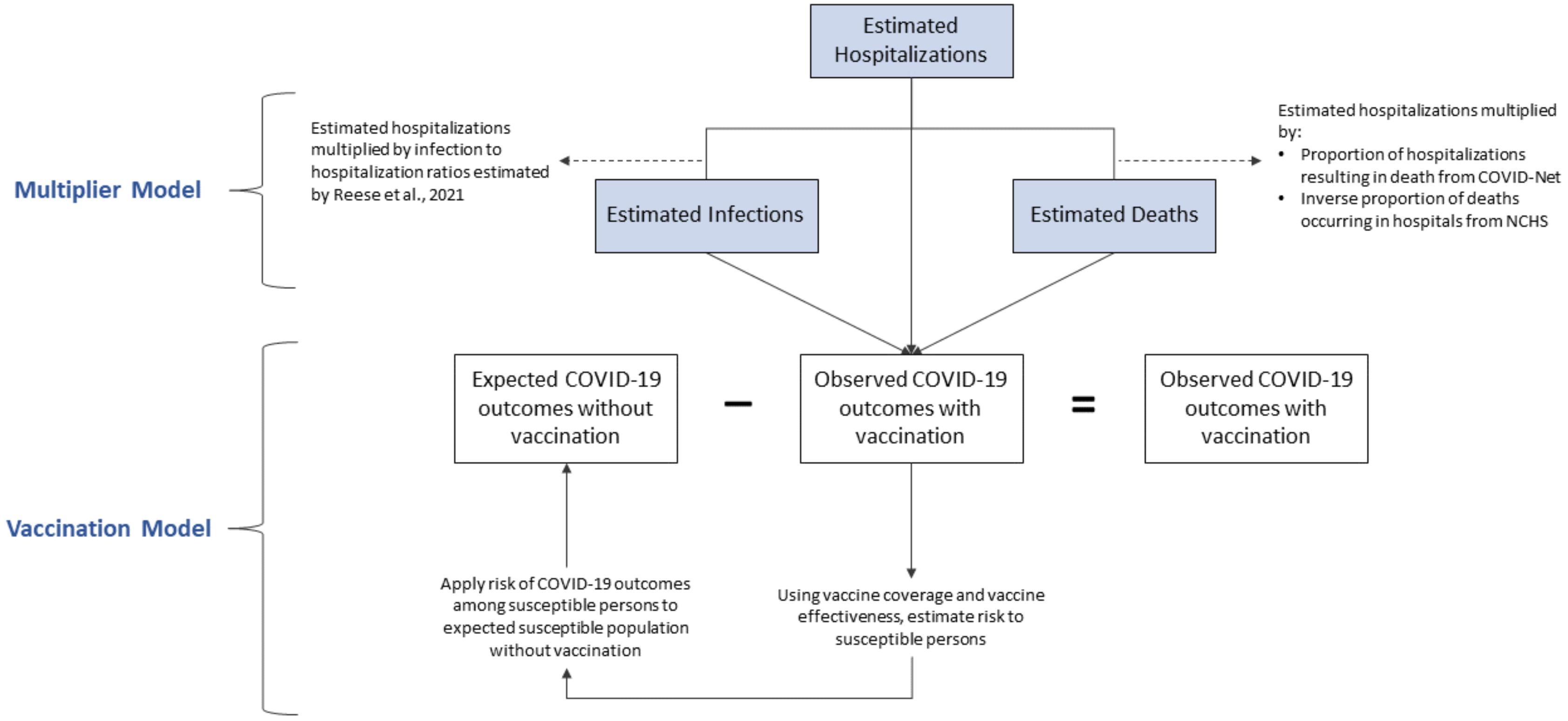

© 2022 Steele MK et al. *JAMA Network Open.*

eFigure 3. Distribution of outcomes in model framework.

Within our model framework, the numbers of hospitalizations and deaths are subsets of the number of infections. The hospitalization estimates consist of those who were hospitalized and did or did not subsequently die. The death estimates consist of those who were previously hospitalized prior to death and those who were never hospitalized.

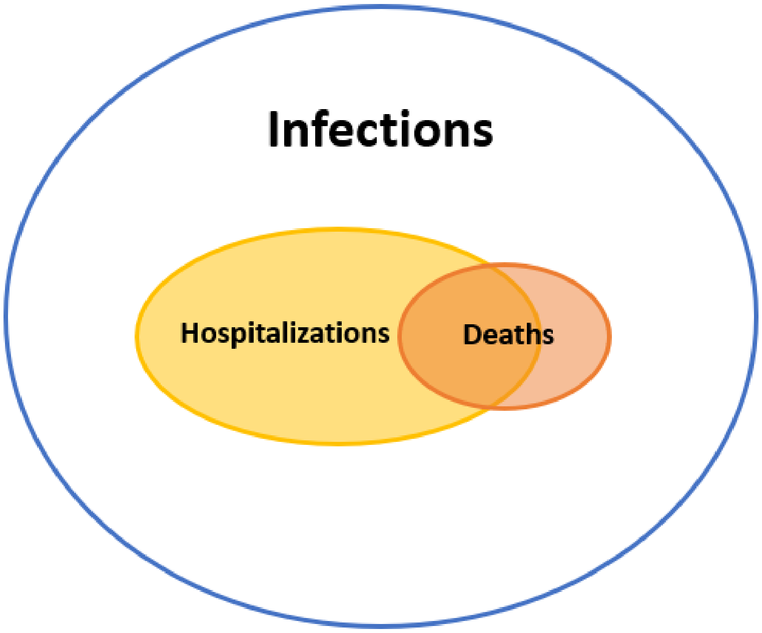

© 2022 Steele MK et al. *JAMA Network Open.*

eFigure 4. Burden prevented model schematic.

A. Compartmental model to estimate changes in susceptible population by infection and vaccination over time. B. Compartmental model to estimate changes in susceptible population over time in the absence of vaccination.

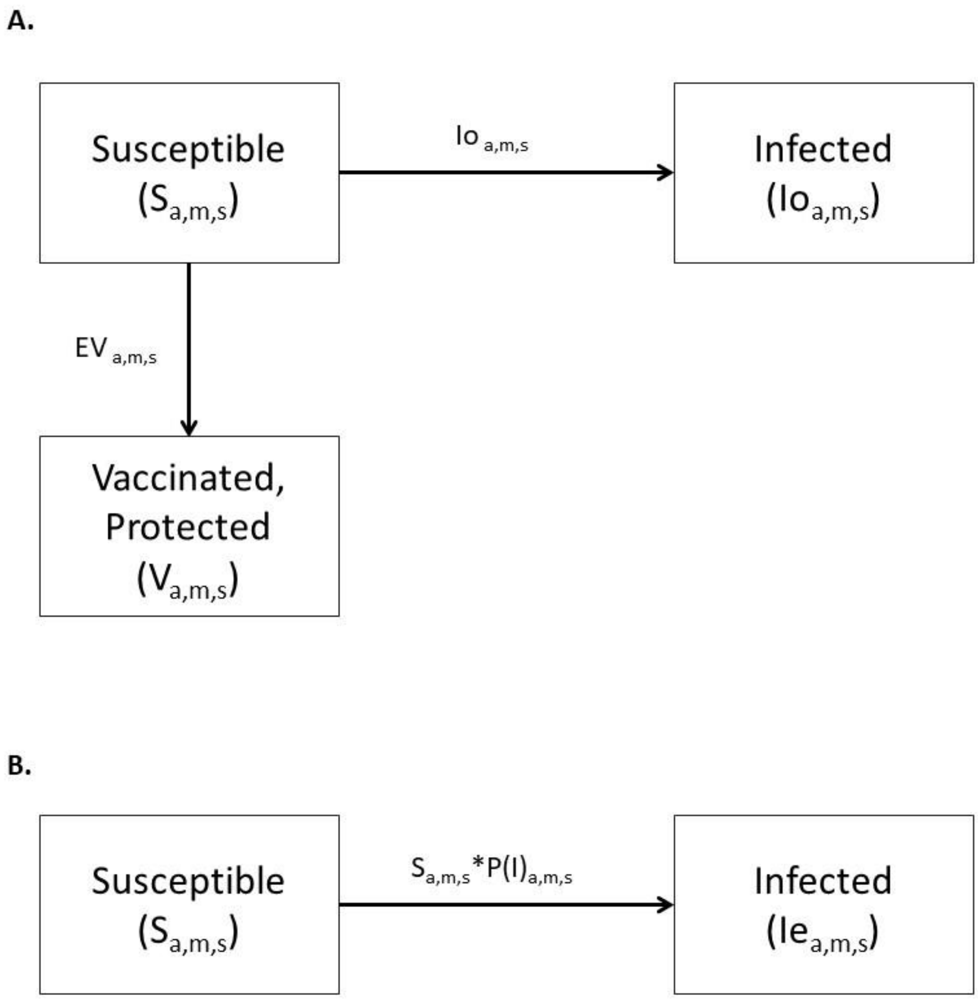

Abbreviations (notation): a= age group, m= month, s=state, S=susceptible, Io= observed infections, EV=effectively vaccinated, V=vaccinated and protected, Ie= expected infections in the absence of vaccination, P(I)=risk of infection among susceptible population.

© 2022 Steele MK et al. *JAMA Network Open.*

eTable 1. Estimates (range) of observed number of COVID-19-associated hospitalizations in the U.S. during December 1, 2020 – September 30, 2021.

We drew 5,000 samples randomly between the 10th and 90th percentiles of 20,000 samples from a posterior distribution of numbers of hospitalizations estimated from a Bayesian hierarchical model. The data presented here represent the median (minimum – maximum) values across the 5,000 samples. State-level data were summed to generate national-level counts of hospitalizations by age group and month

Month	Number of Hospitalizations by Age Group		
	**18-49 years**	**50-64 years**	**65+ years**
December 2020	115,800 (136,000-162,000)	145,300 (171,300-198,500)	179,500 (195,500-215,500)
January 2021	103,700 (120,900-141,600)	126,100 (148,200-173,300)	327,600 (358,100-390,500)
February 2021	59,600 (73,100-89,200)	67,700 (81,600-98,400)	289,800 (319,000-353,100)
March 2021	60,500 (69,900-81,500)	59,900 (68,200-78,000)	131,800 (148,000-166,300)
April 2021	62,700 (71,500-81,900)	57,800 (64,800-72,900)	78,700 (85,100-92,300)
May 2021	42,900 (49,800-58,600)	37,300 (42,200-48,600)	69,000 (74,200-80,300)
June 2021	32,100 (38,100-44,900)	27,100 (32,000-37,200)	48,400 (52,300-57,400)
July 2021	52,800 (65,300-80,300)	44,200 (52,100-63,500)	36,600 (40,400-44,800)
August 2021	98,800 (116,300-138,500)	96,000 (114,200-137,400)	54,600 (61,300-68,000)
September 2021	110,900 (130,500-159,300)	108,600 (129,800-154,300)	158,400 (182,500-209,600)

© 2022 Steele MK et al. *JAMA Network Open.*

eTable 2. Percent of COVID-19 place of death data suppressed.

Percent of state and month strata with suppressed data by age group and by inpatient versus non-inpatient places of death

Age Group	Percent of cells[a] for inpatient setting suppressed	Percent of cells[a] for non-inpatient setting suppressed
18-49	13%	3%
50-64	17%	32%
≥65	26%	46%

a.cells are strata for month and state

© 2022 Steele MK et al. *JAMA Network Open.*

eTable 3. Median proportion (range) of hospitalizations resulting in death by age group and month in the U.S.

Values are estimated from COVID-NET surveillance data.

Month	18-49 years		50-64 years		≥65 years	
	Median	Range in Model	Median	Range in Model	Median	Range in Model
May 2020	0.02	(0.01-0.03)	0.11	(0.07-0.16)	0.24	(0.21-0.26)
June 2020	0.02	(0.01-0.05)	0.07	(0.02-0.16)	0.16	(0.12-0.19)
July 2020	0.02	(0.003-0.07)	0.06	(0.04-0.09)	0.20	(0.17-0.24)
August 2020	0.01	(0.005-0.02)	0.04	(0.01-0.11)	0.18	(0.14-0.23)
September 2020	0.03	(0.005-0.08)	0.05	(0.02-0.10)	0.18	(0.15-0.23)
October 2020	0.03	(0.02-0.06)	0.05	(0.02-0.10)	0.12	(0.09-0.15)
November 2020	0.02	(0.01-0.03)	0.06	(0.04-0.10)	0.16	(0.14-0.18)
December 2020	0.02	(0.01-0.04)	0.09	(0.07-0.12)	0.19	(0.16-0.22)
January 2021	0.04	(0.02-0.08)	0.10	(0.07-0.14)	0.14	(0.11-0.19)
February 2021	0.01	(0.004-0.03)	0.06	(0.04-0.10)	0.09	(0.05-0.14)
March 2021	0.02	(0.01-0.04)	0.07	(0.04-0.10)	0.11	(0.08-0.16)
April 2021	0.03	(0.01-0.08)	0.06	(0.04-0.10)	0.11	(0.08-0.16)
May 2021	0.02	(0.01-0.04)	0.05	(0.03-0.09)	0.12	(0.09-0.15)
June 2021	0.02	(0.01-0.04)	0.06	(0.04-0.09)	0.10	(0.07-0.15)
July 2021	0.03	(0.01-0.08)	0.08	(0.04-0.13)	0.20	(0.16-0.24)
August 2021	0.01	(0.004-0.04)	0.11	(0.07-0.15)	0.17	(0.11-0.26)
September 2021	0.08	(0.05-0.11)	0.14	(0.08-0.22)	0.14	(0.09-0.20)

© 2022 Steele MK et al. *JAMA Network Open.*

eTable 4. Estimated proportion of COVID-19-associated deaths occurring in hospital settings out of all places of death

In our model we used fixed values of the proportion of deaths occurring in hospital settings by age-group, month and state. The table below shows by age group the median estimated proportions and ranges collapsed over time and by state

**Age Group**	**Median proportion of deaths occurring in hospital setting**	**Range in proportion of deaths occurring in hospital setting[a]**
18-49 years	1.00	0.50 – 1.00
50-64 years	0.74	0.20 – 1.00
≥65 years	0.55	0.13 – 1.00

a. Range represents the range in values over time (May 2020 – September 2021) and across 50 states

© 2022 Steele MK et al. *JAMA Network Open.*

eTable 5. Percent of ≥18 year olds that had completed vaccine series by HHS Region and month.

In our model we used fixed values of the percent of the population that had completed vaccine series by age-group, month and state. The table below shows the aggregated percent of ≥18 year olds that had completed vaccine series by HHS region and month

Month	HHS Region[a]									
	1	2	3	4	5	6	7	8	9	10
December 2020	0.01	0.01	0.01	0.01	0.01	0.01	0.01	0.01	0.00	0.01
January 2021	2.82	2.55	2.50	2.42	2.71	3.06	2.70	3.29	2.11	2.75
February 2021	7.25	8.76	8.00	7.39	8.52	8.34	6.84	8.34	8.05	9.15
March 2021	14.24	14.01	12.43	9.95	13.45	11.65	13.90	12.80	13.15	12.53
April 2021	21.07	20.73	19.19	14.85	19.48	16.17	17.27	18.28	17.83	16.96
May 2021	18.01	14.74	13.44	8.24	11.18	8.72	8.17	11.40	12.95	14.75
June 2021	7.50	7.94	6.68	4.62	5.75	5.32	4.25	5.75	6.49	7.29
July 2021	2.35	3.39	2.47	2.44	2.17	2.82	2.25	2.54	3.07	2.50
August 2021	2.52	3.74	2.44	4.10	2.54	4.81	3.67	3.10	3.56	2.57
September 2021	2.38	3.96	2.29	4.39	2.44	4.46	2.94	3.25	3.38	3.37
Cumulative Percent vaccinated[b]	78.14	79.83	69.45	58.41	68.24	65.35	62.00	68.77	70.59	71.87

a. HHS Region 1: Connecticut, Maine, Massachusetts, New Hampshire, Rhode Island, Vermont; Region 2: New Jersey, New York; Region 3: Delaware, Maryland, Pennsylvania, Virginia, and West Virginia; Region 4: Alabama, Florida, Georgia, Kentucky, Mississippi, North Carolina, South Carolina, and Tennessee; Region 5: Illinois, Indiana, Michigan, Minnesota, Ohio, and Wisconsin; HHS Region 6: Arkansas, Louisiana, New Mexico, Oklahoma, and Texas; HHS Region 7: Iowa, Kansas, Missouri, and Nebraska; HHS Region 8: Colorado, Montana, North Dakota, South Dakota, Utah, and Wyoming; HHS Region 9: Arizona, California, Hawaii, Nevada; Region 10: Alaska, Idaho, Oregon, and Washington

b. Cumulative percent of ≥18 year olds that had completed vaccine series (i.e., 2 doses of BNT162b2 or mRNA-1273 vaccines and 1 dose of JNJ-78436735 vaccine) through September 30, 2021.

© 2022 Steele MK et al. *JAMA Network Open.*

eTable 6. Estimates (95% uncertainty interval) of SARS-CoV-2 infections and COVID-19-associated hospitalizations and deaths prevented among COVID-19 vaccinated persons in the U.S during December 1, 2020 - September 30, 2021, assuming fixed, pre-Delta VE over time.

Age Group	Number Prevented (95% Uncertainty Interval)		
	Infections	Hospitalizations	Deaths
18-49 years	14,448,000 (10,536,000-20,177,000)	316,000 (250,000-398,000)	15,000 (7,000-22,000)
50-64 years	8,428,000 (6,025,000-12,778,000)	547,000 (466,000-693,000)	69,000 (42,000-101,000)
65+ years	4,730,000 (3,527,000-6,342,000)	778,000 (629,000-971,000)	158,000 (108,000-221,000)
Total	28,007,000 (22,392,000-36,910,000)	1,650,000 (1,432,000-1,956,000)	242,000 (180,000-318,000)

a. Point estimates for the total outcomes prevented will not necessarily equal to the sum of the age group-specific point estimates. They represent the median total outcomes prevented from 5,000 simulations of the model.

© 2022 Steele MK et al. *JAMA Network Open.*

eReferences

1. Couture A, Iuliano AD, Chang HH, et al. Estimating COVID-19 Hospitalizations in the United States with surveillance data using a Bayesian Hierarchical model. *medRxiv*. Published online October 18, 2021:2021.10.14.21264992. doi:10.1101/2021.10.14.21264992

2. Murphy SL, Xu J, Kochanek KD, Arias E, Tejada-Vera B. Deaths: final data for 2018. *Natl Vital Stat Reports*. 2021;69(13). Accessed November 19, 2021. https://stacks.cdc.gov/view/cdc/100479

3. Reese H, Iuliano AD, Patel NN, et al. Estimated Incidence of Coronavirus Disease 2019 (COVID-19) Illness and Hospitalization—United States, February–September 2020. *Clin Infect Dis*. 2020;72(12):e1010-e1017. doi:10.1093/cid/ciaa1780

4. Tokars JI, Rolfes MA, Foppa IM, Reed C. An evaluation and update of methods for estimating the number of influenza cases averted by vaccination in the United States. *Vaccine*. 2018;36(48):7331-7337. doi:10.1016/j.vaccine.2018.10.026

© 2022 Steele MK et al. *JAMA Network Open.*
